# Atherogenic index of plasma and cardiovascular disease risk in cardiovascular-kidney-metabolic syndrome stage 1 to 3: a longitudinal study

**DOI:** 10.3389/fendo.2025.1517658

**Published:** 2025-02-04

**Authors:** Yu Zhang, Yue Song, Yinfei Lu, Tao Liu, Ping Yin

**Affiliations:** ^1^ Department of Epidemiology and Biostatistics, School of Public Health, Tongji Medical College, Huazhong University of Science and Technology, Wuhan, China; ^2^ Department of Cardiovascular Medicine, Wuhan Red Cross Hospital, Wuhan, China

**Keywords:** CKM syndrome, AIP index, CVD, CHARLS, the elderly

## Abstract

**Background:**

Cardiovascular disease (CVD) remains a major contributor to the global disease burden. Previous studies have established a link between the atherogenic index of plasma (AIP) and CVD. However, it remains unclear whether cumulative AIP and AIP control influence the future incidence of CVD in individuals with Cardiovascular-Kidney-Metabolic (CKM) syndrome. This study aims to explore the association between cumulative AIP, AIP control levels, and the risk of CVD in individuals with CKM syndrome from stages 1 to 3.

**Methods:**

Participants with CKM syndrome were drawn from the China Health and Retirement Longitudinal Study (CHARLS). Cumulative AIP was calculated using triglycerides (TG) and high-density lipoprotein cholesterol (HDL-C), while AIP control levels were categorized into four groups via k-means clustering. CVD was defined by self-reported heart disease or stroke. Multivariable logistic regression and restricted cubic spline analysis were employed to examine the association between AIP and incident CVD in individuals with CKM syndrome.

**Results:**

A total of 793 participants (18.84%) developed CVD. After adjusting for confounders, cumulative AIP were associated with the developing CVD (OR=1.139, 95% CI: 1.017-1.275, P=0.0245). Compared to group 1 (best AIP control), the OR (95% CI) for incident CVD were 1.278 (0.959-1.702) for group 2, 1.329 (1.076-1.641) for group 3, and 1.195 (0.974-1.465) for group 4. Restricted cubic spline regression indicated the relationship between cumulative AIP and CVD risk is linear (P for nonlinear = 0.3377).

**Conclusions:**

In middle-aged and elderly individuals with CKM syndrome, higher cumulative AIP and poorer AIP control were associated with an elevated incidence of CVD. These findings suggest that enhanced assessment of the AIP index could inform targeted prevention strategies for CVD in the context of CKM syndrome.

## 1 Introduction

Cardiovascular disease (CVD) remains the leading cause of morbidity and mortality worldwide, significantly contributing to the global disease burden (1). According to the Global Burden of Disease (GBD) report in 2019, CVD accounted for 18.5 million deaths globally, representing approximately 31% of all deaths (2). In the United States, coronary heart disease caused an estimated 558,000 deaths, while ischemic stroke was responsible for 109,000 deaths (3). In China, the number of CVD-related deaths more than doubled, rising from 2.42 million in 1990 to 4.58 million in 2019 (4). Thus, early identification of individuals at high risk for CVD is critical for preventing disease progression.

Dyslipidemia is a critical contributor to CVD. The relationship between CVD and traditional lipid markers, including total cholesterol (TC), low-density lipoprotein cholesterol (LDL-C), triglycerides (TG), high-density lipoprotein cholesterol (HDL-C), and lipoprotein, has been well established (5). The atherogenic index of plasma (AIP), calculated as the log-transformed ratio of TG to HDL-C in molar concentrations, was first introduced by Dobiásová as a biomarker for plasma atherosclerosis (6). AIP integrates TG and HDL-C levels, not only reflecting the TG-to-HDL-C ratio but also indicating the size of lipoprotein particles, offering a more accurate representation of dyslipidemia’s pathogenicity and specificity compared to high TG or low HDL-C levels alone (7). AIP has emerged as a superior predictor of CVD risk compared to individual lipid markers (8).

In an October 2023 Presidential Advisory by the American Heart Association (AHA), Cardiovascular-Kidney-Metabolic (CKM) syndrome was defined as a systemic disorder driven by complex pathophysiological interactions between metabolic risk factors, chronic kidney disease (CKD), and the cardiovascular system (9). CKM syndrome impacts vascular integrity, promotes atherogenesis, affects myocardial function, disrupts hemostasis, and alters cardiac conduction (9, 10). The CKM stages underscore the pivotal role of excessive and dysfunctional adiposity as a key initiating pathophysiological mechanism, providing an opportunity to identify individuals earlier in their disease trajectory (11).

Indeed, numerous studies in recent years have validated the association between the AIP index and the risk of various conditions, including diabetes, prediabetes, hypertension, metabolic syndrome, myocardial infarction, and non-alcoholic fatty liver disease (12–15). However, the relationship between the AIP index and CVD in individuals with CKM syndrome remains unclear.

Although CVD prevention has been extensively studied over the years, its relation to the CKM population remains insufficiently explored. Given the significant role of CKM syndrome in the progression of cardiovascular disease, it is essential to examine the association between AIP and cardiovascular incidence in individuals with CKM syndrome. Therefore, our study aims to investigate the relationship between AIP control levels, cumulative AIP, and the risk of CVD in individuals with CKM syndrome (from stages 1 to 3) using data from the China Health and Retirement Longitudinal Study (CHARLS).

## 2 Methods

### 2.1 Data source and study population

The data for this study were drawn from the CHARLS, a nationally representative longitudinal survey of individuals aged 45 and older in China. CHARLS adhered to the principles outlined in the Declaration of Helsinki and received approval from the Institutional Review Board at Peking University (IRB00001052-11015). Utilizing a multistage probability sampling method, the baseline survey was conducted between 2011 and 2012 greater than 90% were of Han ethnicity (Wave 1). To date, CHARLS has released data from four subsequent follow-up waves (Wave 2 in 2013, Wave 3 in 2015, Wave 4 in 2018, and Wave 5 in 2020) (16).

**Figure 1:**
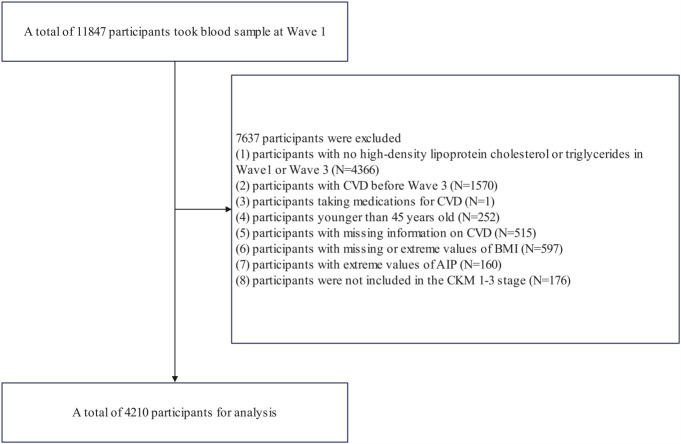
Flowchart of the study population.

The flowchart (**Figure 1**) outlines the inclusion and exclusion criteria for this study. Initially, we excluded 4,366 participants who lacked data on HDL-C or TG in Wave 1 or Wave 3. Additionally, 1,570 participants with pre-existing CVD before Wave 3 and one participant on cardiovascular medications were excluded. We also excluded 252 participants under the age of 45. Further exclusions included 515 participants missing CVD information, 597 participants with missing or extreme body mass index (BMI) values, and 160 participants whose AIP values in 2012 and 2015 were outside the 1st or 99th percentiles. Lastly, 176 participants who did not meet the criteria for CKM syndrome from stage 1 to stage 3 were removed. In total, 4,210 participants were included in the final analysis.

### 2.2 Variables

#### 2.2.1 Calculation of atherogenic index of plasma index

In this study, AIP was defined as follows: AIP = lg [TG (mg/dL)/HDL-C (mg/dL)], and cumulative AIP is calculated as follows: (AIP2012 + AIP2015)/2×(2015 − 2012) (6, 17).

#### 2.2.2 Definition of CKM syndrome stage 0 to 3

The stages of CKM Syndrome, from 1 to 3, are groupified based on the AHA Presidential Advisory Statement on CKM Syndrome. CKM syndrome is defined by a staging framework that begins with stage 1, characterized by excess or dysfunctional adiposity; stage 2, involving metabolic risk factors or moderate to high-risk CKD; and stage 3, marked by subclinical CVD in CKM or risk equivalents of subclinical CVD (such as high-risk CKD or a high predicted risk of CVD) (9). For this groupification, a high predicted 10-year CVD risk, as calculated by the Framingham Risk Score, was used as a risk equivalent for subclinical CVD (18). The estimated glomerular filtration rate was determined using the Chinese Modification of Diet in Renal Disease equation (19).

#### 2.2.3 CVD diagnosis

The primary outcome of this study was the incidence of CVD during the follow-up period (Wave 4 and Wave 5). Consistent with prior research, information regarding a history of heart disease was obtained through the standardized question: “Did your doctor tell you that you have been diagnosed with a heart attack, angina pectoris, coronary heart disease, heart failure, or other heart problem?” The occurrence of stroke was determined using the question: “Did your doctor tell you that you were diagnosed with a stroke?” The CHARLS research team employed stringent quality control measures for data collection and verification to ensure data reliability (16). CVD was defined as self-reported heart disease or stroke.

#### 2.2.4 Data collection

The following data were collected for the purposes of this study:

Demographic data encompassed age, gender, education level, menopause status, and marital status. Body measurements included systolic blood pressure (SBP), diastolic blood pressure (DBP), BMI, and waist circumference. Lifestyle factors comprised smoking status, alcohol consumption, and depression. Data regarding disease and medication history covered hypertension, diabetes, liver diseases, and lung diseases. Laboratory test results included Glycated Hemoglobin A1c (HbA1C), fasting blood glucose (FBG), TG, TC, HDL-c, LDL-c, platelet count (PLT), blood urea nitrogen (BUN), serum creatinine (Scr), C-reactive protein (CRP), and uric acid (UA).

Individuals reporting a history of diabetes or undergoing treatment for this condition, as well as those with a baseline FBG of ≥126 mg/dL or an HbA1c of ≥6.5%, were groupified as having diabetes (20). Depression was assessed using the 10-item short form of the Center for Epidemiologic Studies Depression Scale, with participants scoring ≥10 identified as exhibiting depressive symptoms (21). Other medical statuses were determined through self-reporting.

### 2.3 Handling of missing variables

Certain data were absent from the CHARLS database, and the number and proportion of missing values for each variable are presented in **Supplementary Table S1**. While most variables demonstrated only minimal data incompleteness, multiple imputation was utilized to retain the largest possible sample size, thereby more accurately reflecting the true conditions.

### 2.4 Statistical analysis

K-means clustering is a technique used to identify structural clusters within a dataset, maximizing similarity within clusters and dissimilarity between them (22). In our study, we used k-means clustering to group and the AIP values in each group were presented as means ± standard deviation (SD).

To present the baseline information, for continuous variables exhibiting a normal distribution, statistics were summarized as means ± SD, and differences between groups were analyzed using analysis of variance. In contrast, for continuous variables not conforming to a normal distribution, the median and interquartile ranges were employed for statistical description, with group differences assessed via the Kruskal-Wallis H test. Categorical variables were represented by frequencies and percentages, and intergroup differences were evaluated using the χ² test. The relationship between AIP and the cumulative incidence of CVD was examined using both univariate and multivariate logistic regression models. The crude model was unadjusted for any variables. In Model I, adjustments were made for age and gender. Model II further adjusted the results for current smoking, current drinking, education level, and marital status. Model III included adjustments for age, gender, current smoking, current drinking, education level, marital status, hypertension, diabetes, depression, BUN, Scr, TC, LDL-c, CRP, UA, and PLT. Results from the logistic regression analysis are reported as odds ratios (ORs) with 95% confidence intervals (CIs). To explore the association between AIP and CVD incidence across various demographic characteristics, subgroup analyses were performed based on age groups (< 60 *vs*. ≥ 60 years), gender, menopause status, marital status, smoking status, drinking status, and CKM syndrome stages (Stage 1 to Stage 3). Additionally, to investigate the potential nonlinear relationship between AIP and CVD incidence, restricted cubic spline (RCS) regression was applied to the total population and CKM syndrome stages 1 to 3. Sensitivity analyses were also conducted on data prior to multiple imputation to verify the robustness of the results (**Supplementary Tables S2**, **Supplementary Figure S1**). All statistical analyses were executed using R software (version 4.4.0) and SAS software (version 9.4). A two-sided *P*-value of < 0.05 was considered statistically significant in all calculations.

## 3 Results

### 3.1 K-means clustering of participants

**Figure 2:**
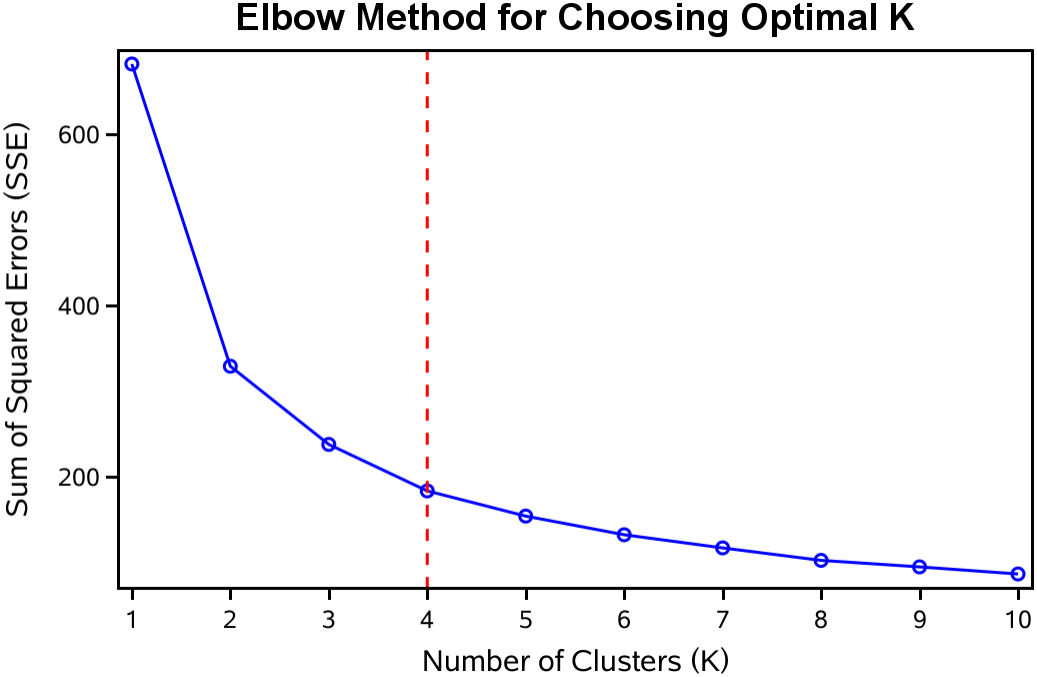
The AIP clustering by k-means clustering.

**Figure 3:**
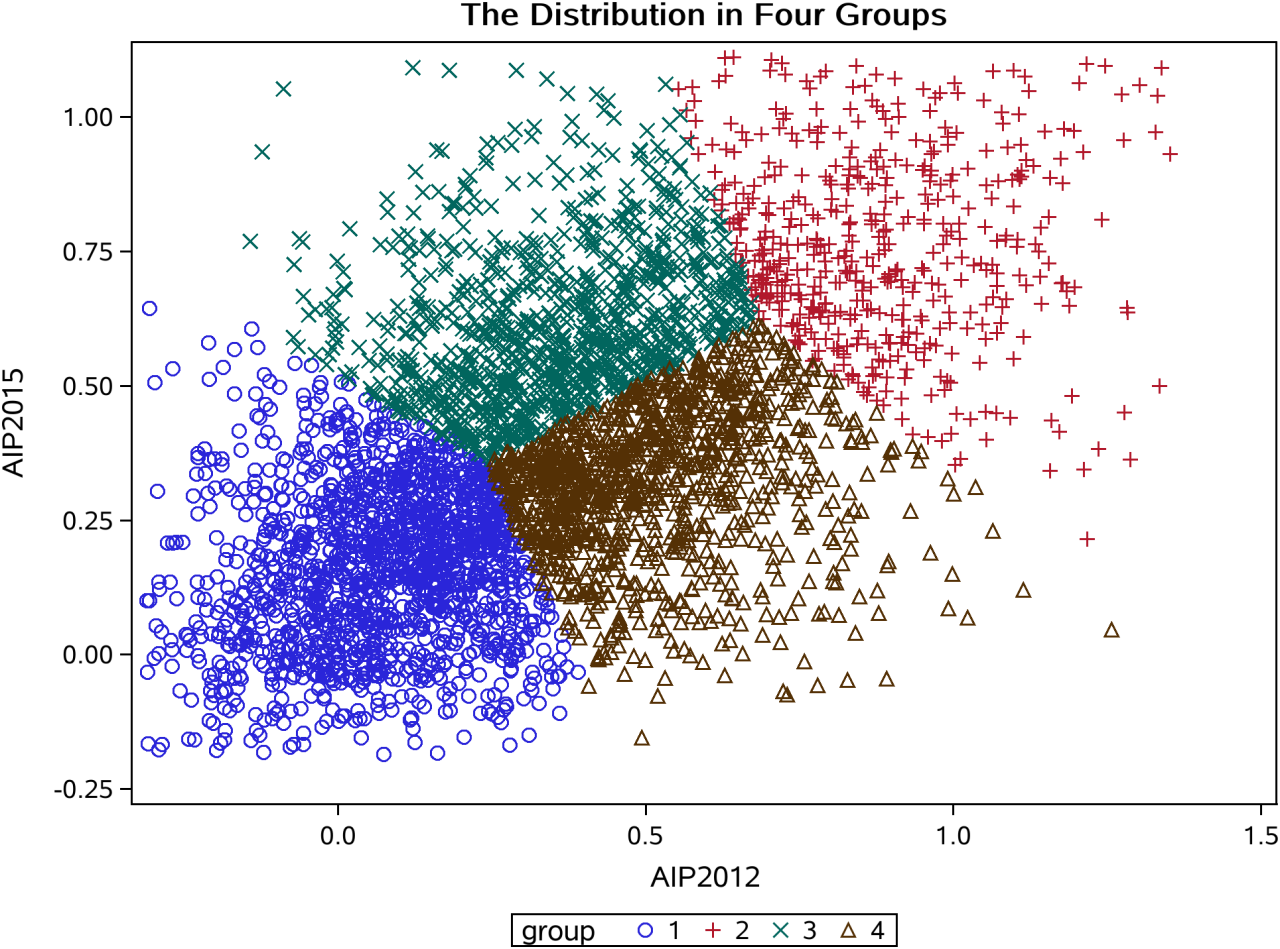
The distribution of AIP clustering by k-means clustering.

In our study, k-means clustering produced optimal results with four clusters (**Figure 2**). The grouping details are illustrated in **Figure 3**. In Group 1, the mean AIP was 0.07 in 2012 and 0.16 in 2015, indicating well-controlled AIP levels. Group 2 had a mean AIP of 0.84 in 2012 and 0.75 in 2015, reflecting persistently high AIP levels with slight improvement. In Group 3, the mean AIP increased from 0.35 in 2012 to 0.61 in 2015, indicating a gradual rise from low to high levels with insufficient control. Finally, in Group 4, the mean AIP decreased from 0.51 in 2012 to 0.32 in 2015, demonstrating a shift from higher to lower levels (**Table 1**).

**Table 1:** Plasma atherogenic index groupified by K-means clustering.

	Group1(N=1551)	Group2(N=491)	Group3(N=1001)	Group4(N=1167)
AIP2012	0.07 ± 0.14	0.88 ± 0.17	0.35 ± 0.16	0.51 ± 0.16
AIP2015	0.16 ± 0.15	0.76 ± 0.17	0.61 ± 0.14	0.32 ± 0.13
cumulative AIP	0.35 ± 0.32	2.45 ± 0.34	1.44 ± 0.36	1.25 ± 0.33

### 3.2 Baseline characteristics of participants

A total of 4,210 participants were enrolled, of whom 55.30% were female, with an average age of 58.73 ± 8.44 years. Among them, 793 participants (18.84%) ultimately developed CVD. Baseline characteristics, as shown in **Table 2**, indicated that compared to Group 1, participants in the other groups exhibited a lower prevalence of current smoking, alcohol consumption, lung diseases, liver diseases, and depression, but a higher prevalence of hypertension and diabetes. In terms of laboratory test data, participants in the other groups had lower levels of BUN and HDL-c but higher levels of FBG, Scr, TC, TG, CRP, and UA compared to Group 1. Furthermore, we provided a baseline description of the missing variables prior to interpolation, which reflects similar findings (**Supplementary Table S2**).

**Table 2:** Baseline characteristics of individuals groupified by plasma atherogenic index measured.

Characteristics	Group1	Group2	Group3	Group4	P-value
N	1551	491	1001	1167	
Age, years	59.00 ± 6.00	57.00 ± 6.00	57.00 ± 6.00	58.00 ± 5.00	<0.0001
Gender, n (%)					0.0442
male	735 (47.39%)	214 (43.58%)	420 (41.96%)	513 (43.96%)	
female	816 (52.61%)	277 (56.42%)	581 (58.04%)	654 (56.04%)	
Married, n (%)	1303 (84.01%)	445 (90.63%)	863 (86.21%)	1011 (86.63%)	0.0874
Education level, n (%)					0.0474
Elementary school or lower	1128 (72.73%)	326 (66.40%)	704 (70.33%)	839 (71.89%)	
Middle school and above	423 (27.27%)	165 (33.60%)	297 (29.67%)	328 (28.11%)	
Waist circumference, cm	80.20 ± 5.20	91.00 ± 5.60	87.00 ± 6.40	86.10 ± 6.70	<0.0001
BMI, kg/m2	21.93 ± 1.93	25.42 ± 2.18	23.98 ± 2.10	23.57 ± 2.11	<0.0001
Systolic, mmHg	123.67 ± 11.33	130.67 ± 11.33	126.67 ± 11.67	128.33 ± 13.00	<0.0001
Diastolic, mmHg	73.00 ± 7.33	78.33 ± 7.33	75.33 ± 7.00	75.33 ± 7.00	<0.0001
Current Smoking, n (%)	606 (39.07%)	176 (35.85%)	372 (37.16%)	415 (35.56%)	0.2570
Current Drinking, n (%)	579 (37.33%)	160 (32.59%)	303 (30.27%)	379 (32.48%)	0.0015
Platelets, (×10^9/L)	203.00 ± 42.00	212.00 ± 49.00	203.00 ± 42.00	208.00 ± 44.00	0.1134
BUN, mg/dl	15.52 ± 2.74	14.82 ± 2.18	15.13 ± 2.60	14.62 ± 2.30	<0.0001
FBG, mg/dl	100.62 ± 7.20	109.26 ± 9.18	102.24 ± 7.56	103.32 ± 7.92	<0.0001
Scr, mg/dL	0.72 ± 0.09	0.77 ± 0.11	0.75 ± 0.10	0.73 ± 0.09	<0.0001
TC, mg/dl	185.95 ± 19.72	197.94 ± 24.36	194.85 ± 24.74	189.82 ± 24.36	<0.0001
TG, mg/dl	71.68 ± 13.27	249.57 ± 46.02	107.97 ± 20.36	139.83 ± 27.44	<0.0001
HDL-c, mg/dl	60.70 ± 8.12	34.41 ± 4.25	48.33 ± 6.19	44.07 ± 5.80	<0.0001
LDL-c, mg/dl	112.11 ± 18.56	104.00 ± 23.20	124.49 ± 22.04	115.21 ± 20.88	<0.0001
CRP, mg/dl	0.76 ± 0.30	1.31 ± 0.63	1.14 ± 0.52	1.02 ± 0.46	<0.0001
HBA1C, %	5.10 ± 0.30	5.20 ± 0.30	5.20 ± 0.30	5.10 ± 0.20	<0.0001
UA, mg/dl	4.04 ± 0.69	4.68 ± 0.79	4.27 ± 0.72	4.23 ± 0.68	<0.0001
Lung diseases, n (%)	154 (9.93%)	33 (6.72%)	80 (7.99%)	94 (8.05%)	0.0852
Liver disease, n (%)	57 (3.68%)	10 (2.04%)	33 (3.30%)	28 (2.40%)	0.1301
Depression, n (%)	585 (37.72%)	139 (28.31%)	315 (31.47%)	417 (35.73%)	0.0002
Hypertension, n (%)	236 (15.22%)	155 (31.57%)	210 (20.98%)	248 (21.25%)	<0.0001
Diabetes, n (%)	151 (9.74%)	137 (27.90%)	133 (13.29%)	208 (17.82%)	<0.0001
CVD, n (%)	252 (16.25%)	100 (20.37%)	212 (21.18%)	229 (19.62%)	0.0088
Heart disease, n (%)	177 (11.41%)	69 (14.05%)	131 (13.09%)	166 (14.22%)	0.1392
Stroke, n (%)	94 (6.06%)	43 (8.76%)	103 (10.29%)	87 (7.46%)	0.0011
CKM stage, n (%)					<0.0001
1	163 (10.51%)	0 (0.00%)	92 (9.19%)	39 (3.34%)	
2	713 (45.97%)	216 (43.99%)	475 (47.45%)	564 (48.33%)	
3	675 (43.52%)	275 (56.01%)	434 (43.36%)	564 (48.33%)	

### 3.3 The relationship between the AIP and the incidence of CVD in a population with CKM syndrome stages 1-3

**Figure 4:**
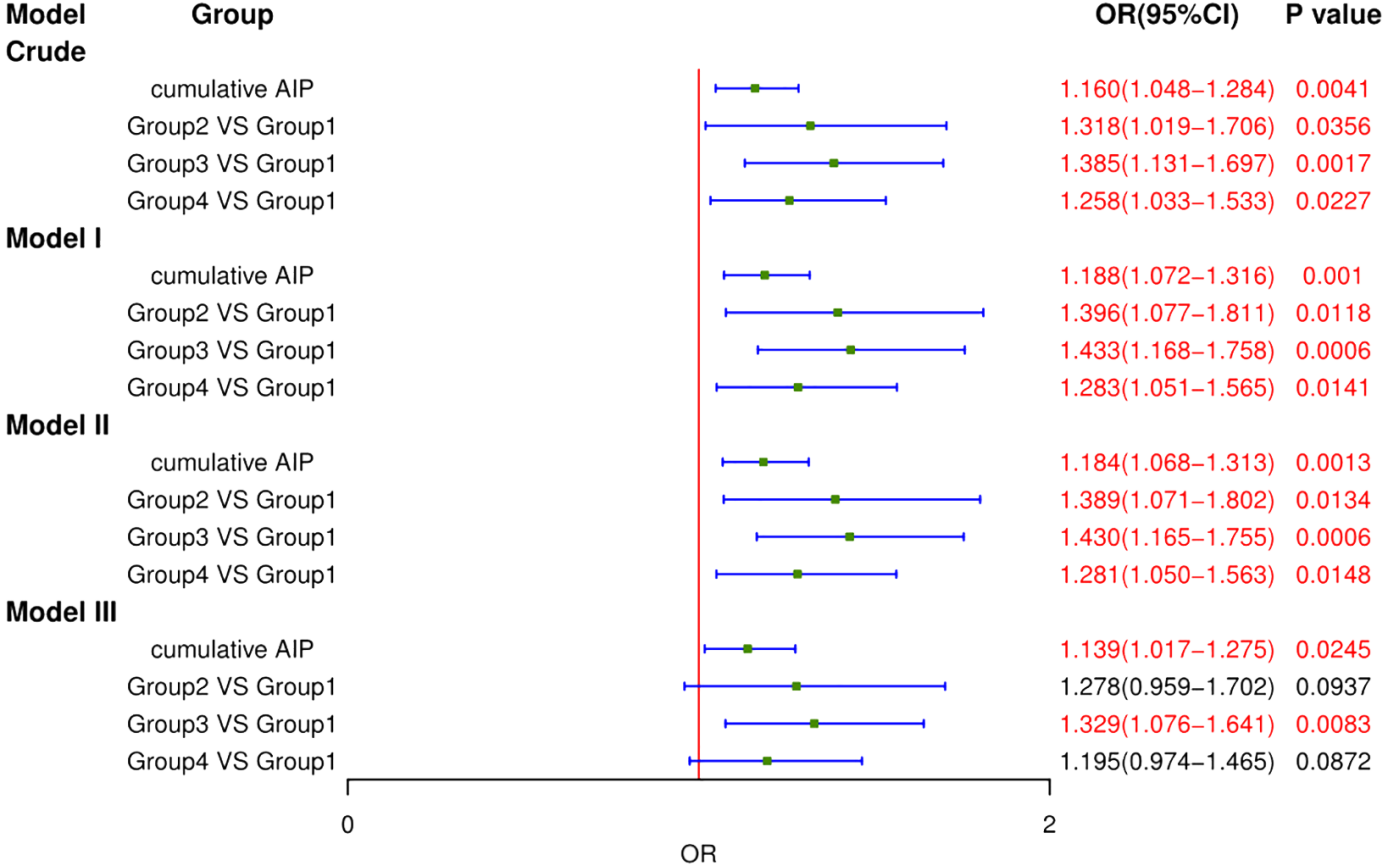
Logistic regression analysis for the association between different groups and CVD. MODEL I: adjusted for Age, Gender. MODEL II: adjusted for Age, Gender, Current Smoking, Current Drinking, Education level, Marital status. MODEL III: adjusted for Age, Gender, Current Smoking, Current Drinking, Education level, Marital status, Hypertension, Diabetes, Depression, BUN, Scr, TC, LDL-c, CRP, UA, PLT.

To evaluate the association between AIP and CVD incidence among participants across CKM syndrome stages 1–3, four logistic regression models were established (**Figure 4**). Initially, the crude model indicated that cumulative AIP were associated with the odds of developing CVD (OR = 1.160, 95% CI: 1.048–1.284, *P* = 0.0041). Model I was consistent with the crude model (OR = 1.188, 95% CI: 1.072–1.316, *P* = 0.0010). Model II maintained this association (OR = 1.184, 95% CI: 1.068–1.313, *P* = 0.0013). After correcting for multiple confounders, Model III also showed the same trend (OR = 1.139, 95% CI: 1.017–1.275, *P* = 0.0245).

To further elucidate the relationship between AIP and CVD incidence, participants were stratified into four groups using K-means clustering. In the fully adjusted Model III, the OR (95% CI) for incident CVD were 1.278 (0.959–1.702) for Group 2, 1.329 (1.076–1.641) for Group 3, and 1.195 (0.974–1.465) for Group 4, compared to Group 1. Group 3 indicated that the deterioration of AIP is significantly correlated with increased CVD incidence. While the results for Group 2 and Group 3 were not statistically significant, they nonetheless demonstrated a discernible trend toward heightened risk of CVD incidence. Furthermore, we evaluated the association of AIP and CVD without multiple filling, which were consistent with our findings (**Supplementary Figure S1**).

**Figure 5:**
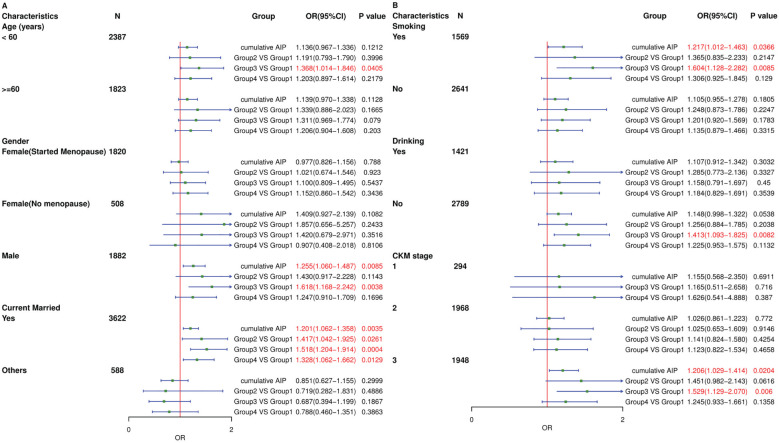
Subgroup analysis of the associations between different groups and CVD. The model was adjusted for Age, Gender, Current Smoking, Current Drinking, Education level, Marital status, Hypertension, Diabetes, Depression, BUN, Scr, TC, LDL-c, CRP, UA, PLT. **(A)** Subgroup analyses were performed based on age, gender and marital status. **(B)** Subgroup analyses were performed based on smoking, drinking and CKM syndrome stages.

To investigate the relationship between AIP and CVD incidence, subgroup analyses were conducted across various age groups, genders, marital statuses, smoking statuses, drinking statuses, and CKM syndrome stages (Stage 1 to Stage 3). As presented in **Figures 5A, B**, the subgroup analysis of cumulative AIP revealed a higher risk of CVD among males, currently married individuals, smokers, and patients in CKM Stage 3 compared to other subgroups. Relative to Group 1, Group 3 exhibited an increased risk of CVD in males, individuals under 60, currently married individuals, smokers, non-drinkers, and those in CKM Stage 3.

**Figure 6:**
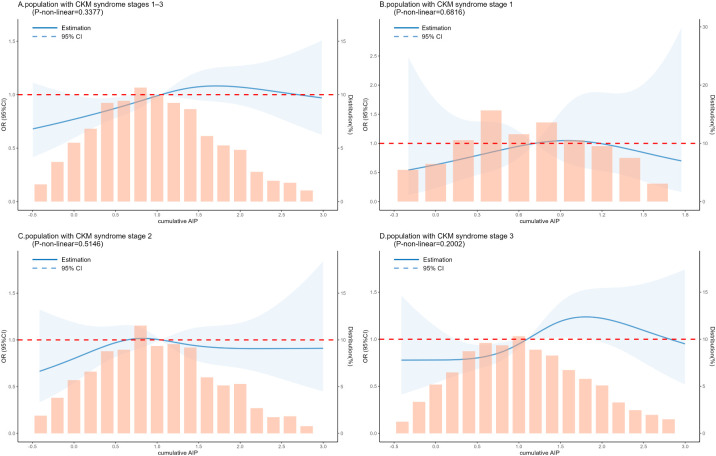
RCS analysis between the cumulative AIP index and CVD incidence in a population with CKM syndrome stages 1–3. The model was adjusted for Age, Gender, Current Smoking, Current Drinking, Education level, Marital status, Hypertension, Diabetes, Depression, BUN, Scr, TC, LDL-c, CRP, UA, PLT. **(A)** Population with CKM syndrome stages 1–3. **(B)** Population with CKM syndrome stage 1. **(C)** Population with CKM syndrome stage 2. **(D)** Population with CKM syndrome stage 3.

In the restricted cubic spline regression models illustrated in **Figure 6A**, the relationship between cumulative AIP and CVD risk was linear. Similarly, as depicted in **Figures 6B–D**, this linear association persisted across the subgroups of CKM syndrome stages 1 to 3.

## 4 Discussion

Based on the results of a literature search, this study represents the first investigation into the association between the AIP index and CVD within the context of CKM syndrome. Previous studies have demonstrated a significant correlation between the AIP index and CVD in the general population (23–25). Given the interactions among metabolic risk factors, CKD, and the cardiovascular system, coupled with the high prevalence of poor CKM health, exploring the relationship between AIP and CVD in the framework of CKM syndrome is deemed crucial (9, 26). In this study, we conducted logistic regression analyses among the elderly Chinese population to assess the relationship between the AIP index and CVD. Through comprehensive analyses of CHARLS datasets, elevated levels of cumulative AIP and inadequate control of AIP exhibited a risk effect on CVD in individuals with CKM syndrome. These findings hold the potential to inform effective prevention strategies for CVD.

AIP serves as a robust marker for predicting the risk of atherosclerosis and coronary heart disease, reflecting the true relationship between protective and atherogenic lipoproteins, as well as correlating with the size of pre- and anti-atherogenic lipoprotein particles (6, 27). Moreover, AIP is believed to more effectively indicate plasma atherogenicity than individual lipid values, given the complex interactions within lipoprotein metabolism (28). The results of ten observational studies conducted in China, Turkey, and South Korea demonstrated that elevated AIP values may be independently associated with the odds of coronary artery disease (29). However, most prior studies assessed AIP at a single time point and overlooked the longitudinal variations of AIP. In our study, we comprehensively considered the longitudinal results of AIP and observed cumulative AIP were associated with CVD (OR=1.139, 95% CI: 1.017-1.275), which aligns with previous findings.

The population with CKM syndrome, characterized by pathophysiological interactions among metabolic risk factors, CKD, and the cardiovascular system, may experience multiorgan dysfunction and adverse cardiovascular outcomes (9). However, previous studies have largely overlooked this high-risk group. Following the Presidential Advisory issued by the AHA, an increasing number of studies have begun to support this concept and concentrate on the CKM population. One cohort study demonstrated a positive linear association between triglyceride glucose-body mass index and increased CVD incidence among individuals with CKM syndrome (30). Additionally, a review highlighted how dysregulation of the RAS during pregnancy and lactation contributes to CKM characteristics in offspring, elucidating the underlying mechanisms (31). Furthermore, a study found that a higher stage of CKM syndrome correlates with an elevated risk of all-cause mortality, particularly pronounced among younger adults (32). Research on CKM syndrome will aid in identifying individuals at high risk for morbidity and mortality, enabling the initiation of preventive strategies before damage occurs.

Our results indicated a trend but revealed no significant statistical correlation between cumulative AIP and the incidence of CVD in CKM syndrome stages 1 and 2. This may be attributed to the absence of significant metabolic or cardiovascular risk factors in these individuals, or to an insufficient sample size, necessitating further research. In contrast, among individuals with heightened metabolic risk factors in CKM syndrome stage 3, the association between cumulative AIP and CVD incidence was significantly amplified. This finding underscores the need to prioritize this population due to their elevated metabolic and cardiovascular risks, aligning with previous studies. Moreover, the results of the RCS analysis further substantiate the linear nature of this association. These findings illuminate the predictive value of cumulative AIP within the CKM syndrome population, facilitating more accurate identification of high-risk individuals.

It is noteworthy that our results underscore the significance of controlling the AIP index. Group 3 and 4 exhibited comparable AIP levels in 2012, with AIP worsening in Group 3 while remaining stable in Group 4. The findings indicated that the deteriorating group had a higher odds ratio (1.329 *vs*. 1.195) compared to the controlled group. This suggests that even when participants presented elevated baseline AIP levels, a reduction in AIP following intervention corresponded with a decreased risk of subsequent CVD. Evidence indicates that a diminished AIP index was associated with a reduction in metabolic syndrome scores and the prevalence of metabolic syndrome (33). However, additional high-quality prospective trials are required to validate our findings.

The exact mechanism linking the AIP and incident CVD in individuals with CKM syndrome remains unclear; however, several underlying biological processes may provide insight. Systemic inflammation is widely recognized as a central factor in the pathogenesis of CVD (34). The AIP is derived from the ratio of TG to HDL-C, and it can be influenced by fasting serum TG levels and inflammatory mediators—a mechanism that has been closely associated with inflammation and oxidative stress, as reported in previous studies (35, 36). Elevated TG levels can damage the vascular endothelium, inducing endothelial dysfunction, while also promoting coagulation and triggering inflammatory responses (28). HDL-C plays a critical role in reverse cholesterol transport and offers cardioprotection through its anti-inflammatory and antioxidant effects (37). Additionally, CKM syndrome impairs vascular integrity, atherosclerotic development, myocardial function, hemostasis, and cardiac conduction, processes intimately linked to TG, HDL-C, and inflammatory mediators (9). Consequently, further investigation into the mechanisms linking AIP and incident CVD in the CKM population is essential to advancing strategies for optimal cardiovascular health.

This study possesses several notable strengths. To our knowledge, it is the first investigation to analyze the AIP index in relation to the risks of incident CVD within the context of the CKM population. We assessed the AIP index as both categorical and continuous variables, comprehensively examining cumulative AIP and its longitudinal changes. Additionally, we utilized data from large-scale national longitudinal surveys and adjusted for multiple confounders, thereby illuminating the intrinsic relationship between AIP control levels and the incidence of new-onset CVD in participants with CKM syndrome. Furthermore, we discovered that elevated AIP and high cumulative AIP significantly increase the incidence of cardiovascular events, which can be conveniently prevented through readily obtainable biochemical parameters.

However, our study has limitations. Firstly, it exclusively included middle-aged and elderly Chinese individuals, and the diagnosis of CVD was self-reported, which may restrict the generalizability of the findings. As understanding of health issues and agreement on diagnoses can vary across different groups of older patients, utilizing medical record data to verify self-reports would provide a more reliable approach (38, 39). Secondly, in defining subclinical CVD, we employed the Framingham 10-year cardiovascular risk score instead of other latest equations, although the former has been validated and widely used in Asian populations (40). Lastly, due to the limited sample size and potential confounding factors that may have been overlooked, the accuracy and stability of the relationship between AIP and new-onset CVD in participants with CKM syndrome may be compromised.

## 5 Conclusions

In conclusion, we had established a correlation between the AIP and an increased incidence of CVD among individuals in CKM syndrome stages 1-3. Therefore, AIP should be considered a straightforward indicator of CVD, and individuals with elevated AIP levels, particularly those in CKM syndrome stage 3, should prioritize CVD prevention. These findings could have profound implications for informing CVD prevention strategies within the context of CKM syndrome.

## Data Availability

The datasets presented in this study can be found in online repositories. The names of the repository/repositories and accession number(s) can be found here: http://charls.pku.edu.cn.
