# Supplementary material for: Atherogenic index of plasma and cardiovascular disease risk in cardiovascular-kidney-metabolic syndrome stage 1 to 3: a longitudinal study
Source: Front Endocrinol (Lausanne). 2025 Feb 4;16:1517658. doi: 10.3389/fendo.2025.1517658 (PMC11832398; doi:10.3389/fendo.2025.1517658)
Supplement: Supplementary file 1 [file DataSheet1.docx]

Supplementary Material

# Supplementary Figures and Tables

## Supplementary Figures

**
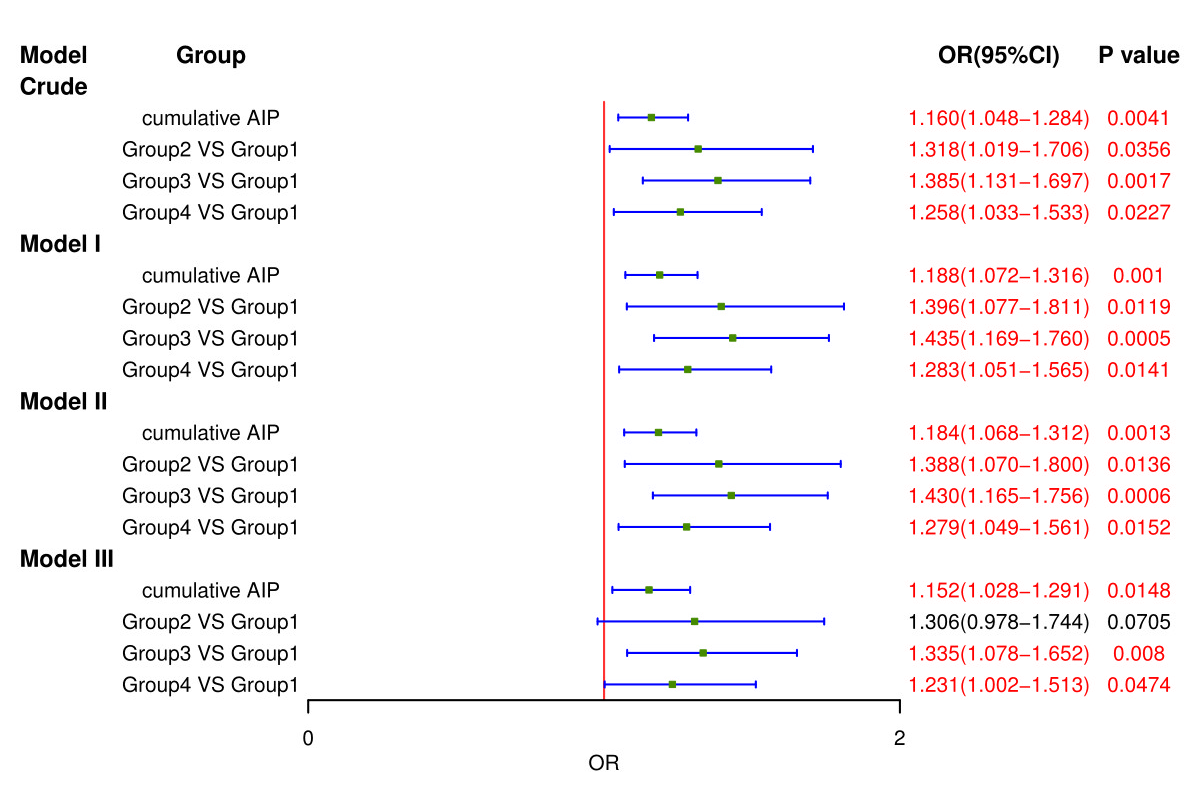
**

**Fig. S1** Logistic regression analysis for the association between different groups and CVD before multiple interpolation

MODEL I: adjusted for Age, Gender

MODEL II: adjusted for Age, Gender, Current Smoking, Current Drinking, Education level, Marital status

MODEL III: adjusted for Age, Gender, Current Smoking, Current Drinking, Education level, Marital status, Hypertension, Diabetes, Depression, BUN, Scr, TC, LDL-c, CRP, UA, PLT.

## Supplementary Tables

**Table S1** Missing number for variables.

| Variables | Missing number (%) |
| --- | --- |
| Age | 0 (0.00%) |
| Gender | 1 (0.02%) |
| Married | 0 (0.00%) |
| Education level | 1 (0.02%) |
| Waist circumference | 7 (0.17%) |
| BMI, kg/m2 | 0 (0.00%) |
| Systolic, mmHg | 36 (0.86%) |
| Diastolic, mmHg | 39 (0.93%) |
| Current Smoking | 0 (0.00%) |
| Current Drinking | 0 (0.00%) |
| Platelets | 69 (1.64%) |
| BUN | 0 (0.00%) |
| FBG | 4 (0.10%) |
| Scr | 4 (0.10%) |
| TC | 2 (0.05%) |
| TG | 0 (0.00%) |
| HDL-c | 0 (0.00%) |
| LDL-c | 1 (0.02%) |
| CRP | 1 (0.02%) |
| HBA1C | 26 (0.62%) |
| UA | 1 (0.02%) |
| Lung diseases, n (%) | 8 (0.19%) |
| Liver disease, n (%) | 17 (0.40%) |
| Depression, n (%) | 24 (0.57%) |
| Hypertension, n (%) | 18 (0.43%) |
| Diabetes, n (%) | 0 (0.00%) |

**Table S2** Baseline Characteristics of missing variables before multiple interpolation.

| Characteristics | Group1 | Group2 | Group3 | Group4 | P-value |
| --- | --- | --- | --- | --- | --- |
| N | 1551 | 491 | 1001 | 1167 |  |
| Age, years | 59.00±6.00 | 57.00±6.00 | 57.00±6.00 | 58.00±5.00 | <0.0001 |
| Gender, n (%) |  |  |  |  | 0.0462 |
| male | 735(47.39%) | 214(43.58%) | 420(42.00%) | 513(43.96%) |  |
| female | 816(52.61%) | 277(56.42%) | 580(58.00%) | 654(56.04%) |  |
| Married, n (%) | 1303(84.01%) | 445(90.63%) | 863(86.21%) | 1011(86.63%) | 0.0874 |
| Education level, n (%) |  |  |  |  | 0.0474 |
| Elementary school or lower | 1128(72.73%) | 326(66.40%) | 704(70.33%) | 839(71.89%) |  |
| Middle school and above | 423(27.27%) | 165(33.60%) | 297(29.67%) | 328(28.11%) |  |
| Waist circumference, cm | 80.20±5.20 | 91.00±5.60 | 87.00±6.40 | 86.10±6.70 | <0.0001 |
| BMI, kg/m^2^ | 21.93±1.93 | 25.42±2.18 | 23.98±2.10 | 23.57±2.11 | <0.0001 |
| Systolic, mmHg | 123.83±11.50 | 130.67±11.33 | 126.67±12.00 | 128.33±13.17 | <0.0001 |
| Diastolic, mmHg | 73.00±7.33 | 78.33±7.33 | 75.33±7.00 | 75.33±7.33 | <0.0001 |
| Current Smoking, n (%) | 606(39.07%) | 176(35.85%) | 372(37.16%) | 415(35.56%) | 0.2570 |
| Current Drinking, n (%) | 579(37.33%) | 160(32.59%) | 303(30.27%) | 379(32.48%) | 0.0015 |
| PLT, (×10^9/L) | 203.50±43.50 | 212.00±50.00 | 202.00±42.00 | 207.00±44.00 | 0.1126 |
| BUN, mg/dl | 15.52±2.74 | 14.82±2.18 | 15.13±2.60 | 14.62±2.30 | <0.0001 |
| FBG, mg/dl | 100.62±7.20 | 109.26±9.18 | 102.24±7.56 | 103.32±7.92 | <0.0001 |
| Scr, mg/dL | 0.72±0.09 | 0.77±0.11 | 0.75±0.10 | 0.73±0.09 | <0.0001 |
| TC, mg/dl | 185.95±19.72 | 197.94±24.36 | 194.85±24.74 | 189.82±24.36 | <0.0001 |
| TG, mg/dl | 71.68±13.27 | 249.57±46.02 | 107.97±20.36 | 139.83±27.44 | <0.0001 |
| HDL-c, mg/dl | 60.70±8.12 | 34.41±4.25 | 48.33±6.19 | 44.07±5.80 | <0.0001 |
| LDL-c, mg/dl | 112.11±18.56 | 104.00±23.20 | 124.49±22.04 | 115.21±20.49 | <0.0001 |
| CRP, mg/dl | 0.76±0.30 | 1.31±0.63 | 1.14±0.52 | 1.02±0.46 | <0.0001 |
| HBA1C, % | 5.10±0.30 | 5.20±0.30 | 5.20±0.30 | 5.10±0.20 | <0.0001 |
| UA, mg/dl | 4.04±0.69 | 4.68±0.79 | 4.27±0.72 | 4.23±0.68 | <0.0001 |
| Lung diseases, n (%) | 154(9.94%) | 33(6.73%) | 80(8.00%) | 94(8.08%) | 0.0867 |
| Liver disease, n (%) | 57(3.69%) | 10(2.05%) | 33(3.30%) | 28(2.41%) | 0.1356 |
| Depression, n (%) | 584(37.65%) | 139(28.31%) | 312(31.17%) | 413(35.39%) | 0.0001 |
| Hypertension, n (%) | 235(15.23%) | 155(31.83%) | 210(21.00%) | 248(21.34%) | <0.0001 |
| Diabetes, n (%) | 151(9.74%) | 137(27.90%) | 133(13.29%) | 208(17.82%) | <0.0001 |
| CVD, n (%) | 252(16.25%) | 100(20.37%) | 212(21.18%) | 229(19.62%) | 0.0088 |
| Heart disease, n(%) | 177(11.41%) | 69(14.05%) | 131(13.09%) | 166(14.22%) | 0.1392 |
| Stroke, n(%) | 94(6.06%) | 43(8.76%) | 103(10.29%) | 87(7.46%) | 0.0011 |
| CKM stage, n (%) |  |  |  |  | <0.0001 |
| 1 | 163(10.51%) | 0(0.00%) | 92(9.19%) | 39(3.34%) |  |
| 2 | 713(45.97%) | 216(43.99%) | 475(47.45%) | 564(48.33%) |  |
| 3 | 675(43.52%) | 275(56.01%) | 434(43.36%) | 564(48.33%) |  |
